# Revolutionizing Dentistry by Exploring the Potential of Cone-Beam Computed Tomography: A Review

**DOI:** 10.7759/cureus.80368

**Published:** 2025-03-10

**Authors:** Mrunali G Gharat, Amit Patil, Aarti S Bedia, Himmat Jaiswal, Saudamini More

**Affiliations:** 1 Dentistry, Bharati Vidyapeeth (Deemed to Be University) Dental College and Hospital, Navi Mumbai, IND; 2 Conservative Dentistry and Endodontics, Bharati Vidyapeeth (Deemed to Be University) Dental College and Hospital, Navi Mumbai, IND; 3 Oral Medicine and Radiology, Bharati Vidyapeeth (Deemed to Be University) Dental College and Hospital, Navi Mumbai, IND; 4 Public Health Dentistry, Bharati Vidyapeeth (Deemed to Be University) Dental College and Hospital, Navi Mumbai, IND

**Keywords:** ai, cbct, dental imaging, diagnostic accuracy, endodontics, orthodontics, periodontology, prosthodontics, radiation exposure, specialized training

## Abstract

Cone-beam computed tomography (CBCT) is a groundbreaking dental imaging technique that produces three-dimensional, high-resolution images of the oral cavity. It has several benefits over conventional imaging techniques, including as better visualization of intricate anatomical structures, increased diagnostic precision, and reduced radiation exposures in comparison to traditional computed tomography (CT). Numerous dental specialties, including endodontics, orthodontics, prosthodontics, oral surgery, periodontology, and forensic odontology, heavily rely on CBCT. The diagnostic capabilities of CBCT are being improved by recent developments in artificial intelligence (AI), which makes it a useful tool for identifying dental diseases. Longer scan periods, more radiation exposure, and the requirement for specific training for effective interpretation are some of CBCT's drawbacks. The objective of this review is to examine the main clinical uses of CBCT in different dental specialties and assess its benefits over conventional imaging methods in terms of accuracy, precision, and patient outcomes. All things considered, even though CBCT has many advantages, clinical practice must carefully weigh its dangers and limits.

## Introduction and background

The idea of cone-beam computed tomography (CBCT) was introduced to radiology shortly after the first computed tomography (CT) scanner was developed. In 2001, the radiography method known as CBCT was introduced to the United States dental market [1]. Based on flat panel detectors, contemporary dental CBCT scanners have the capability of multimodal imaging, including three-dimensional CBCT imaging as well as two-dimensional panoramic and cephalometric imaging. There are many different gantry geometries, scan technique options, detector sizes, and fields of view for dental CBCT scanners. While some devices provide a variety of selectable X-ray procedures, others function at a single-set X-ray tube voltage [2].

In dentistry, CBCT is a commonly used imaging technique. It makes it possible to see high-contrast, high-quality images of the oral region's bone, teeth, and air cavities. Nowadays, pre-operative implant planning is the main application for CBCT, which is frequently used to evaluate bone quality [3]. A well-established method for precisely quantifying and finding anatomic structures, CBCT imaging is a potent device that enables the diagnosis of three-dimensional structures. Given the extensive use of CBCT technology and the capacity to view the mouth's circumferential areas, there has been a natural expectation that bone abnormalities and furcations will be easier to identify and more readily evident as a patient education tool. Practitioners looking to improve patient diagnosis and care have shown a great deal of interest in CBCT. CBCT has significantly enhanced the visualization of intricate anatomical structures while also optimizing workflows by minimizing the necessity for multiple imaging sessions. This technology allows dental professionals to identify conditions that traditional X-rays may overlook, such as concealed infections, bone deterioration, and the exact positioning of impacted teeth. Additionally, its use in pre-surgical planning provides a more detailed perspective of a patient's anatomy, which can help mitigate risks during procedures like dental implants, root canals, and surgical extractions. However, the broader adoption of CBCT in dentistry presents certain challenges, including concerns regarding radiation exposure, financial implications, and the requirement for specialized training. Therefore, the purpose of this review is to examine the main clinical uses of CBCT in different dental specialties and assess the benefits of CBCT over conventional imaging methods in terms of accuracy, precision, and patient outcomes.

## Review

Methods

**Figure 1:**
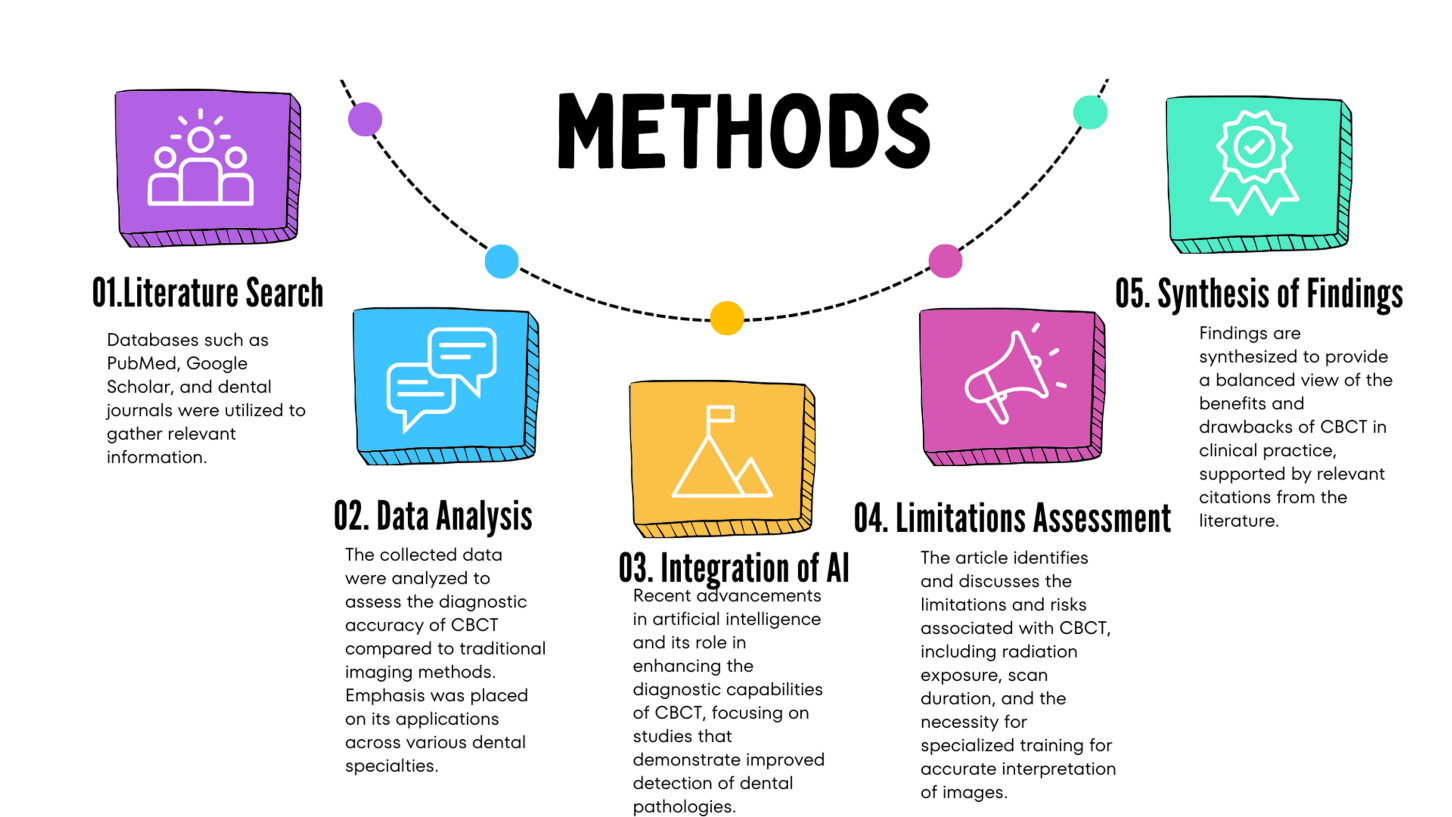
Methods used in conducting this study. CBCT, cone-beam computed tomography; AI: artificial intelligence Image credits: It was created by Mrunali G. Gharat and approved by Amit Patil.

A total of 36 articles were used for writing this review article. This article assesses the uses and efficiency of CBCT in dentistry using a thorough literature review methodology (Figure 1).

Differences Between CT and CBCT

**Figure 2:**
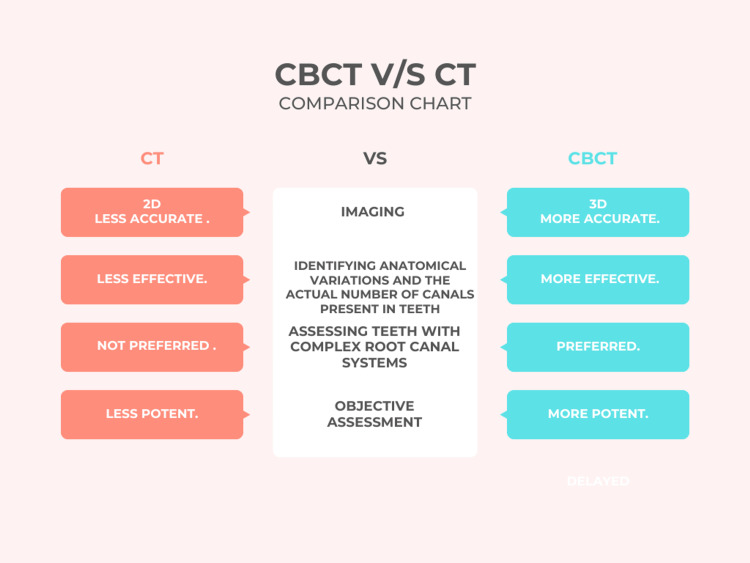
Differences between CT and CBCT. CBCT, cone-beam computed tomography; CT, computed tomography Image credits: It was created by Mrunali G. Gharat and approved by Amit Patil.

The main differences between CT and CBCT have been compared as shown in Figure 2.

Working of CBCT

**Figure 3:**
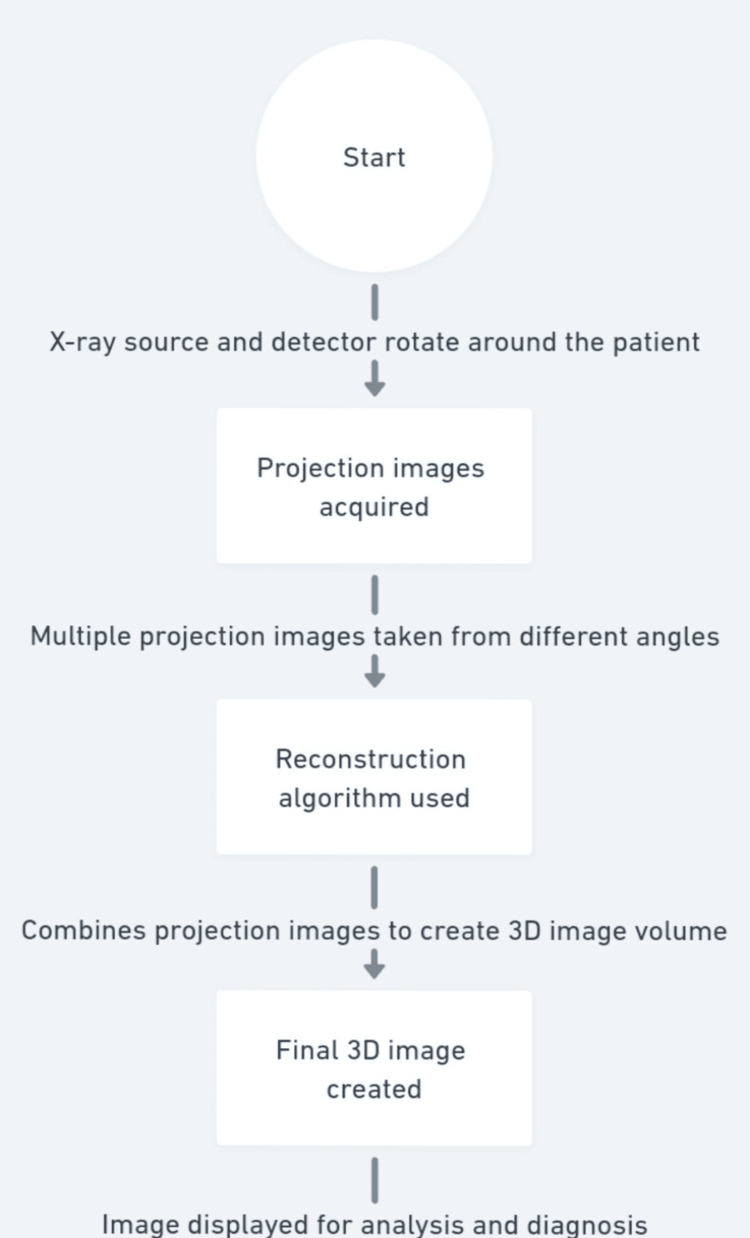
Working of CBCT. CBCT, cone-beam computed tomography Image credits: It was created by Mrunali G. Gharat and approved by Amit Patil.

The working of CBCT has been shown in Figure 3.

Integration of CBCT With AI

**Figure 4:**
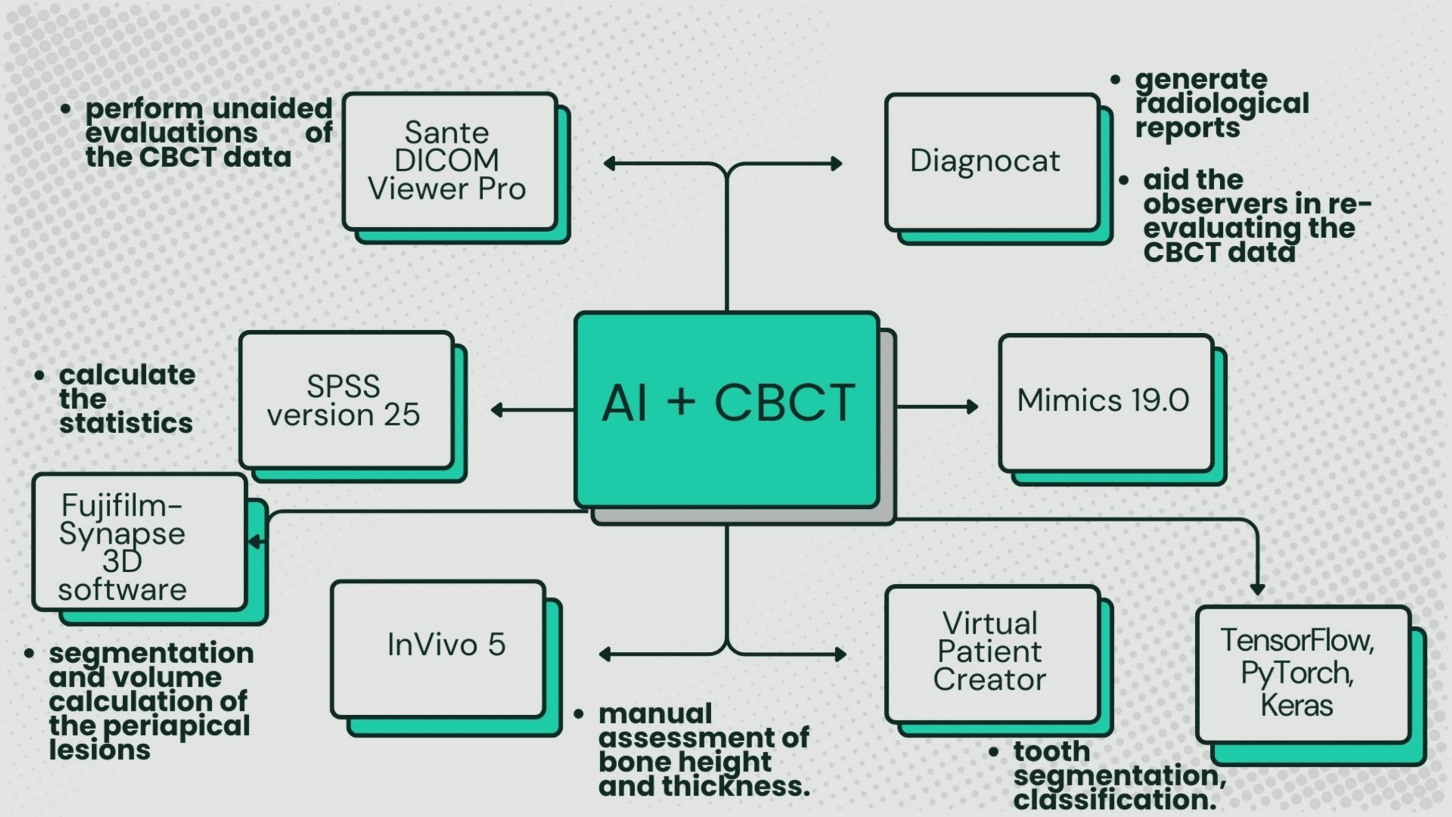
Integration of CBCT with AI. CBCT, cone-beam computed tomography; DICOM, Digital Imaging and Communications in Medicine; SPSS, Statistical Package for the Social Sciences; AI, artificial intelligence Image credits: It was created by Mrunali G. Gharat and approved by Amit Patil.

Recent research has shown how artificial intelligence (AI) might improve the diagnostic capabilities of CBCT in dentistry. When it comes to identifying different dental pathologies, such as caries, periodontal diseases, and periapical lesions, AI systems have demonstrated performance that is on par with or better than that of human specialists [4]. While maintaining high accuracy, these AI-based methods can drastically cut down on the amount of time needed for picture analysis [5]. Additionally, adding denoising modules to AI systems can enhance reader interpretability and diagnostic performance, especially in places with a shortage of qualified clinicians [6]. In addition to making stand-alone interpretation easier, the combination of CBCT and AI improves the quality and effectiveness of dental diagnostics by acting as an efficient decision-support system [4]. The various AI tools that are integrated with CBCT and their use have been shown in Figure 4. These developments imply that AI-assisted CBCT analysis may develop into a crucial instrument in dentistry, possibly enhancing patient outcomes and treatment.

Applications of CBCT in dentistry

Endodontics

CBCT, which provides three-dimensional visualization of the craniofacial skeleton with lower radiation doses than standard CT, has become a useful imaging tool in endodontics [7]. By offering undistorted pictures, CBCT improves treatment planning, increases the diagnosis of endodontic illnesses, and circumvents the drawbacks of conventional radiography [7,8]. In endodontics, it is used for diagnosis, treatment planning, and post-treatment assessment [9]. The exponential growth in publications on the subject over the previous 20 years is indicative of the growing usage of CBCT in endodontics. Even though CBCT has the potential for a number of endodontic uses, more study is required to develop evidence-based recommendations for its utilization in clinical settings [7].

Orthodontics

CBCT, which offers three-dimensional imaging for diagnosis and treatment planning, has become more important in the field of orthodontics [10]. Although CBCT provides better diagnostic information than traditional radiography, its usage should be justified because it exposes users to more radiation [11]. In orthodontics, CBCT is frequently used to evaluate airways, temporomandibular joint abnormalities, impacted teeth, and craniofacial abnormalities [11,12]. Planning orthognathic surgery and identifying the best locations for temporary skeletal anchoring devices are two further uses for it [11]. To assess the risk-benefit ratio of CBCT in comparison to traditional imaging, prospective randomized clinical trials are necessary [13]. Experts stress that CBCT should only be utilized in cases where it offers fresh information that can enhance therapy execution or change treatment plans, rather than on a regular basis [12].

Prosthodontics

Beyond its use in implant rehabilitation, CBCT offers a number of uses and advantages in maxillofacial prosthodontics. Implant planning, denture therapy, and temporomandibular joint imaging are just a few of its many uses in prosthodontics. According to John et al., CBCT facilitates overdenture therapy, template-assisted maxillofacial repair, and precise movement simulation [14]. Compared to two-dimensional imaging, it enables more accurate diagnosis, treatment planning, and outcome analysis [15].

Oral Surgery

CBCT is a useful radiographic diagnostic technique that enables precise three-dimensional planning and evaluation in oral and maxillofacial surgery. Dentoalveolar surgery, dental implants, temporomandibular joint problems, orthognathic surgery, trauma, and pathology are just a few of the domains in which it finds use [16,17]. CBCT improves operative precision by supporting preoperative evaluation, surgical planning, and postoperative analysis. Oral and maxillofacial pathology, maxillofacial trauma, temporomandibular joint disorders, cleft palate, orthognathic and diagnostic fractures and inflammatory conditions of the jaws and sinuses, surgical planning for impacted teeth, implant surgeries, cysts, and tumors are the main uses of CBCT in Oral and Maxillofacial surgery [18]. Nevertheless, CBCT has drawbacks, including insufficient soft tissue representation for assessing specific infections and clinical states. Even though CBCT is widely used, it might not always be a substitute for other imaging modalities [16,17].

Periodontology

In cases of periodontal abnormalities, especially in maxillary molars with furcation involvement, CBCT can enhance diagnostic precision and maximize treatment planning. According to Walter et al., CBCT is now advised for use in periodontology mainly in difficult cases, particularly those involving maxillary molars, where its advantages in diagnostic and treatment planning exceed any potential disadvantages [19]. The diagnosis of periodontal disease primarily relies on clinical signs and symptoms. However, when bone destruction is present, radiographs serve as important diagnostic tools to complement the clinical evaluation. In two-dimensional imaging, the assessment of bone craters, lamina dura, and the periodontal bone level is restricted due to projection geometry and the overlapping of nearby anatomical structures. These constraints of two-dimensional radiographs can be addressed by utilizing three-dimensional imaging methods, such as CBCT, which produces three-dimensional volumetric images and is widely employed in dentistry. This is crucial for diagnosing intra-bony defects, furcation involvement, and buccal/lingual bone loss. While CBCT presents significant advantages in periodontics, it should be employed only when appropriate, taking into account both the need for the examination and the potential risks involved [20].

Forensic Odontology

CBCT, which offers many benefits over conventional radiography, has become a useful tool in forensic odontology. Rapid, non-invasive, affordable, and radiation-free three-dimensional imaging is possible with CBCT [21]. Bite mark analysis, facial reconstruction, sex determination, and age estimation are some of its uses in forensic dentistry [21,22]. CBCT is especially helpful for comparative identification and age assessment using pulp narrowing techniques since it can generate high-quality three-dimensional pictures of teeth and jaws [21]. The benefits of the technique include digital measurement capabilities, optical enlargement, and immediate picture capturing [23]. However, there are still issues, such as the requirement for appropriate training to handle artifacts and technological constraints [22]. Although CBCT research in forensic odontology is still in its infancy in India, it has the potential for use in the future [24].

Limitations of CBCT

The limitations of CBCT have been shown along with the studies describing them in Table 1. According to some of the studies conducted, CBCT was not superior in terms of lowering patient morbidity compared to 2D imaging, and it also does not seem to affect overall treatment success or duration.

**Table 1:** Limitations of CBCT according to studies conducted. CBCT, cone-beam computed tomography; RCT, randomized controlled trial; CT, computed tomography

Author	Title	Study type	Limitations
[25]	A multicenter, randomized, controlled study examined the clinical utility of CBCT in the extraction of the mandibular third molar.	RCT	For the removal of the mandibular third molar, CBCT is not superior to panoramic radiography in terms of lowering patient morbidity or inferior alveolar nerve damage.
[26]	The effect of using CBCT in the diagnosis of canine impaction and its impact on the orthodontic treatment outcome.	RCT	CBCT improved the diagnosis and treatment of maxillary canine impaction compared to two-dimensional imaging but did not affect overall treatment success or duration.
[27]	Neurosensory abnormalities following surgical excision of the mandibular third molar as determined by CBCT scanning or panoramic imaging: an RCT, or randomized controlled trial.	RCT	Prior to the removal of the mandibular third tooth, CBCT does not lessen neurosensory abnormalities in comparison to panoramic imaging.
[28]	Cytotoxicity and mutagenicity in individuals exposed to ionizing radiation.	RCT	While CBCT exposure does not produce mutagenicity, it can cause cytotoxicity in buccal mucosa cells.
[29]	CBCT in the evaluation of mandibular third molars prior to surgery.	RCT	When compared to panoramic radiography, the use of CBCT for mandibular third molar extractions does not lessen nerve damage or other problems.
[30]	Contrasting conventional CT guidance with CBCT with fluoroscopic overlay for percutaneous abdominopelvic abscess drain placement	RCT	With shorter treatment timeframes, CBCT guidance is comparable to traditional CT guidance for the percutaneous installation of an abscess drain.

Advantages and disadvantages of CBCT

**Figure 5:**
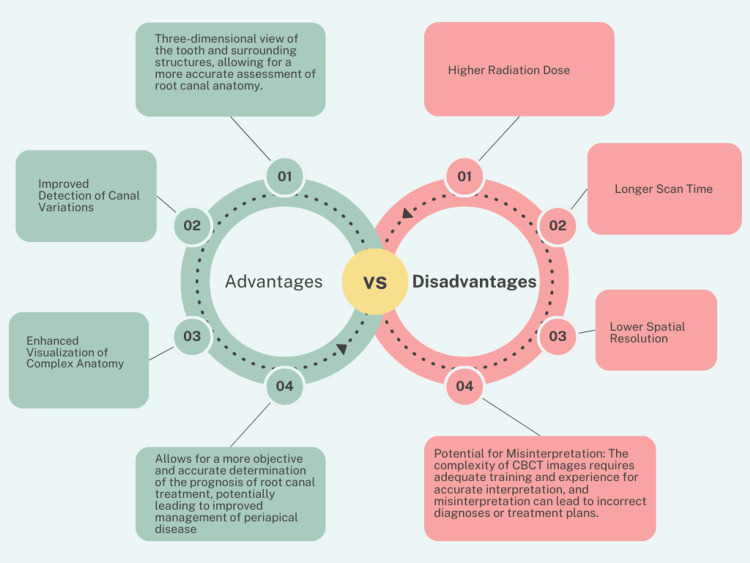
Advantages and disadvantages of CBCT. CBCT, cone-beam computed tomography Image credits: It was created by Mrunali G. Gharat and approved by Amit Patil.

The advantages and disadvantages of CBCT are shown in Figure 5. The majority of the advantages of CBCT are due to its 3D imaging, whereas its disadvantages include higher radiation doses, longer scanning times, and so on.

CBCT in patient education and communication

Tim Joda et al. created a novel application technique that effectively illustrated the viability of combining images from IOS, CBCT, and EOS to create a craniofacial virtual model in three-dimensional static settings. CBCT is a diagnostic imaging method that generates Digital Imaging and Communications in Medicine (DICOM) data by using radiation. To guarantee the best outcomes for patients and healthcare professionals, this data must be carefully interpreted [31]. In healthcare, patient education is essential, and initiatives have been made to enhance provider education and communication, especially in oncology settings [32]. With an emphasis on enhancing the communication climate, patient happiness, and the necessity of long-term implementation, training programs have been created to improve healthcare providers' communication and patient education abilities [33]. In dentistry, CBCT has been used to evaluate the precision and repeatability of landmark recognition in cephalograms, with encouraging outcomes for patients undergoing orthognathic surgery [34]. The significance of precision and consistency in CBCT imaging is further highlighted by the proposal of standardized techniques to measure the variation in voxel value distribution in patient-simulated CBCT data sets [35]. Furthermore, the potential for better treatment planning and delivery has been demonstrated by the development of automated techniques to mimic CBCT-guided treatments in radiotherapy planning systems [36]. All things considered, the research indicates that CBCT contributes significantly to patient education and communication by offering comprehensive imaging information that can support diagnosis, treatment planning, and patient education. Research and development efforts in the healthcare industry are still focused on improving the accuracy and repeatability of CBCT imaging as well as healthcare providers' communication skills.

## Conclusions

Since its inception, CBCT has revolutionized dental imaging by providing high-resolution, three-dimensional views that greatly improve treatment planning and diagnostic accuracy in a variety of dental specialties. It is a vital tool in contemporary dentistry because of its capacity to identify dental diseases and visualize intricate anatomical features. Its diagnostic skills are further enhanced by the incorporation of AI, which expedites the analysis process and enhances results. However, it is important to recognize that CBCT has drawbacks, such as higher radiation exposure, longer scan times, and the need for specific training to properly interpret the pictures. The capability of producing high-resolution, three-dimensional images of craniofacial structures has transformed the approach practitioners take toward intricate procedures, leading to better patient outcomes and facilitating more effective interventions. Although CBCT presents some challenges, such as radiation exposure, financial considerations, and the requirement for specialized training, its benefits significantly surpass these drawbacks, establishing it as a vital element in modern dental practice. As technological advancements progress, the significance of CBCT in dentistry is expected to grow, enhancing the precision, efficiency, and safety of dental treatments for patients globally. These elements emphasize the significance of a well-rounded strategy in clinical practice, where the advantages and disadvantages of CBCT are evaluated. In the end, even though CBCT is a huge improvement in dental imaging, its safe and efficient application in patient care depends on careful thought and adherence to best practices.
